# Uncovering Prognostic Biomarkers Underlying Hepatocellular Carcinoma Through Integrative Multi-Omics and a Network-Based Approach

**DOI:** 10.3390/ijms27104164

**Published:** 2026-05-07

**Authors:** Arshad Husain Rahmani, Anam Beg, Tarique Sarwar, Amjad Ali Khan

**Affiliations:** 1 Department of Medical Laboratories, College of Applied Medical Sciences, Qassim University, Buraydah 51452, Saudi Arabia; t.sarwar@qu.edu.sa; 2 Department of Computer Science, Faculty of Natural Sciences, Jamia Millia Islamia, New Delhi 110025, India; anamrazabeg@gmail.com; 3 Department of Basic Health Sciences, College of Applied Medical Sciences, Qassim University, Buraydah 51452, Saudi Arabia

**Keywords:** Hepatocellular carcinoma, WGCNA, PPIN, cancer, prognosis

## Abstract

Hepatocellular carcinoma (HCC) remains a leading cause of cancer-related mortality worldwide, underscoring the need for robust molecular biomarkers to improve prognosis and therapeutic strategies. Although advances have been made in imaging, surgery, as well as systemic therapies, the prognosis of HCC remains poor due to late detection, high recurrence, and molecular heterogeneity, underscoring the significance of identifying robust prognostic biomarkers and therapeutic targets. mRNA-sequencing data from the TCGA-HCC cohort were examined to recognize differentially expressed genes (DEGs) between tumor and normal tissues. Weighted gene co-expression network analysis (WGCNA) was applied to uncover key gene modules and hub genes. Protein–protein interaction network (PPIN) construction and modular analysis further refined candidate genes. Univariate overall survival (OS) analysis identified five genes (*TTK*, *CENPA*, *NUF2*, *KIF2C*, and *CDCA8*) whose elevated expression significantly correlated with poor patient survival. Pathway enrichment analysis exhibited a strong association with mitotic checkpoint and kinetochore signaling pathways. Mutational profiling demonstrated frequent genomic alterations, particularly in *NUF2*, whereas immune infiltration analysis demonstrated significant correlations between *NUF2* expression and multiple immune cell populations. In this study, we employed an integrative transcriptomic and systems biology approach to recognize prognostically relevant hub genes in HCC. Collectively, this finding highlights the critical genes that may serve as prognostic biomarkers and potential therapeutic targets in HCC.

## 1. Introduction

HCC is the most prevalent primary liver malignancy and a major contributor to global cancer-related morbidity and mortality [1]. HCC is the sixth most common cancer globally and a leading cause of cancer-related mortality, often arising from chronic liver disease, particularly cirrhosis, with increasing risk factors including metabolic dysfunction and alcohol-related liver disease [2,3,4]. The etiology of HCC is multifactorial, with significant contributions from viral infections (hepatitis B and C), alcohol consumption, and metabolic disorders [5,6]. Treatment options vary based on tumor stage and liver function, but challenges remain in achieving effective management [7]. Despite significant advances in diagnostic imaging, surgical resection, and systemic therapies, the long-term prognosis of HCC patients remains poor, primarily due to late-stage diagnosis, high recurrence rates, and pronounced molecular heterogeneity. Therefore, identifying reliable molecular biomarkers that can improve prognostic stratification and uncover novel therapeutic targets is of critical importance.

A powerful method for systematically analyzing transcriptomic changes in cancer is high-throughput RNA-seq [8]. Comprehensive investigations of tumor-specific gene expression patterns across several cancer types, including HCC, have been made possible by large-scale programs like TCGA. DEA of RNA-seq data enables the discovery of genes dysregulated during carcinogenesis; however, conventional single-gene methods often fall short of capturing the intricate regulatory networks that control cancer development. Systems biology-based strategies, particularly WGCNA [9], provide a robust framework for identifying biologically meaningful gene modules based on co-expression patterns. By focusing on gene-gene interactions rather than isolated expression changes, WGCNA facilitates the discovery of functionally coherent gene clusters and central “hub” genes that may play critical roles in disease pathophysiology. Integration of WGCNA with PPINs further enhances the identification of key regulatory nodes within molecular networks.

Hepatocarcinogenesis is characterized by dysregulation of cell cycle progression, mitotic checkpoints, and chromosomal segregation, which is intimately associated with genomic instability and tumor aggressiveness. Numerous investigations into HCC have identified hub genes linked to the cell cycle, such as *CCNA2* and *GSK3B*, through integrated transcriptomic and network analyses, emphasizing their prognostic significance in early HCC and possible roles in disease progression [10,11]. Genes related to spindle assembly, kinetochore function, and mitotic surveillance have been increasingly linked to the development of HCC and unfavorable clinical outcomes. Through combined transcriptome and network-based studies, a team of researchers also discovered hub genes linked to the cell cycle in HCC, demonstrating their potential as prognostic biomarkers and improving future diagnosis and treatment approaches [12]. Additional investigation has revealed that the cell cycle and drug catabolism are the main genes associated with prognosis in HCC.

Many transcriptome markers for HCC prediction have been reported thus far, but their clinical use is still constrained by substantial inter-patient heterogeneity and a lack of functional synchronization. Many current gene sets are produced from wide differential expression without considering the underlying protein-protein interactome, which results in the co-identification of “passenger” genes with actual biological drivers. Additionally, there is a knowledge gap on how chromosomal instability markers affect the tumor-immune interface because few signatures have merged co-expression stability with immune infiltration dynamics, even though the mitotic machinery is a known feature of HCC [13,14,15]. While various computational frameworks have been developed to integrate protein–protein interaction networks (PPINs) into disease module detection—ranging from early graph-based clustering to more recent multi-omic diffusion methods—our study builds upon these established principles by specifically coupling WGCNA-derived co-expression modules with refined modular analysis of PPINs. This integrative approach allows for the filtration of transcriptomic noise and the identification of robust, biologically functional hub genes specifically within the context of the HCC mitotic checkpoint and kinetochore signaling pathways [16,17].

Nevertheless, there is still a lack of a comprehensive analysis that incorporates transcriptome dysregulation, co-expression networks, protein connections, survival relevance, mutational status, and relationships with the TME. In this study, we used an integrative computational pipeline to identify prognostically significant hub genes in HCC using TCGA RNA-seq data. To identify important co-expression modules and hub genes, non-trait-based WGCNA was applied to differentially expressed genes between tumor and normal tissues. PPIN construction, OS analysis, pathway enrichment, mutational profiling, and tumor immune infiltration assessment were used to further evaluate these candidates. To provide insights into potential prognostic biomarkers and therapeutic targets, our comprehensive strategy aimed to identify robust molecular signatures associated with HCC progression and patient survival.

## 2. Results

### 2.1. mRNA-Seq Data Extraction and DEA

**Figure 1:**
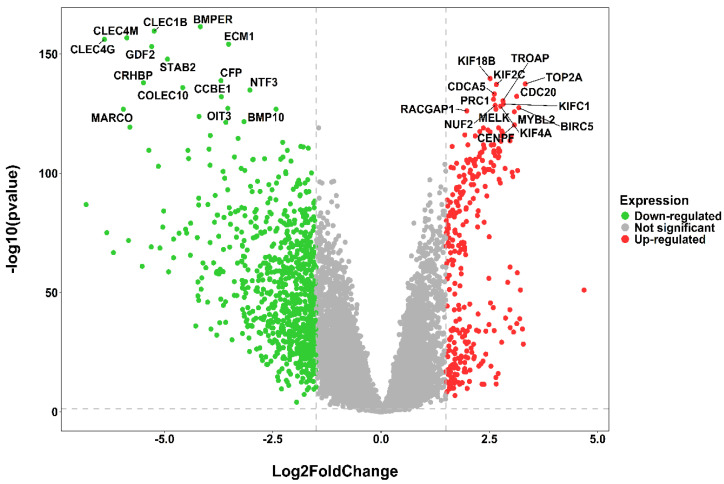
Distribution of 1134 HCC-specific DEGs (green-colored dots specify underexpression and red-colored dots specify overexpression) and nonsignificant genes (gray colored dots) as a volcano plot. Names of the top 15 most underexpressed and the top 15 most overexpressed DEGs are highlighted.

**Figure 2:**
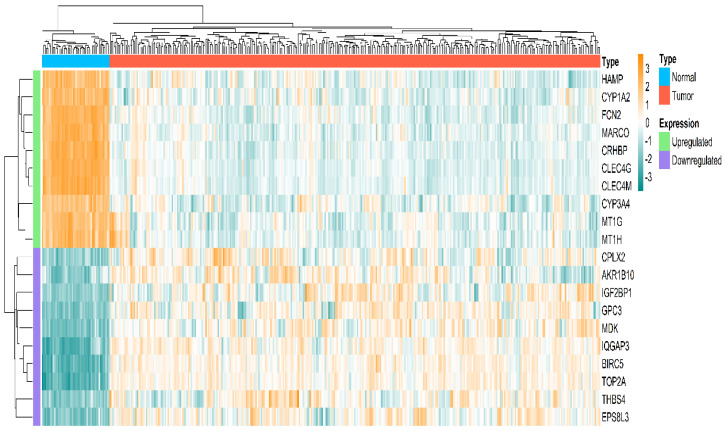
Heatmap plot exhibiting the expression distribution of the top 10 overexpressed and the top 10 underexpressed HCC-specific DEGs across normal and tumor samples.

The HCC-specific mRNA dataset included 413 patient samples, consisting of 363 tumor samples and 50 healthy normal samples. Post pre-processing, duplicate gene handling, we obtained a total of 1134 DEGs via limma package corresponding to *p*-value < 0.05 and $\left|{log}_{2}(fold\;change)\right|>1.5$. Among all the DEGs, 296 were overexpressed and 838 were underexpressed. A volcano plot summarizing the significant and non-significant genes in the TCGA-HCC cohort is displayed in Figure 1. Figure 2 presents a heatmap of the top 10 overexpressed and top 10 underexpressed HCC-specific DEGs.

### 2.2. Non-Trait-Based WGCN Construction and Hub Module/Genes Selection

**Figure 3:**
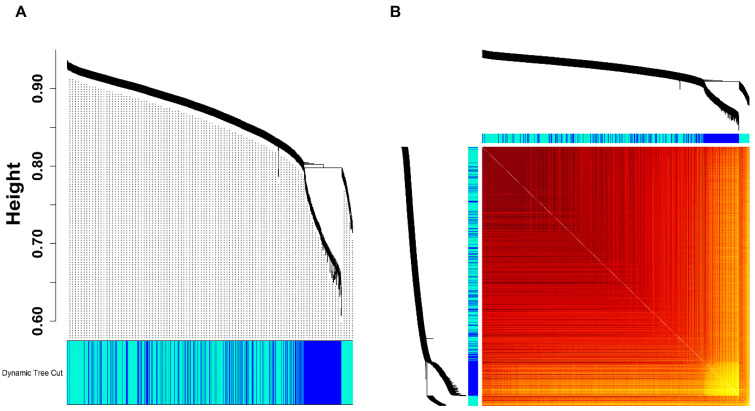

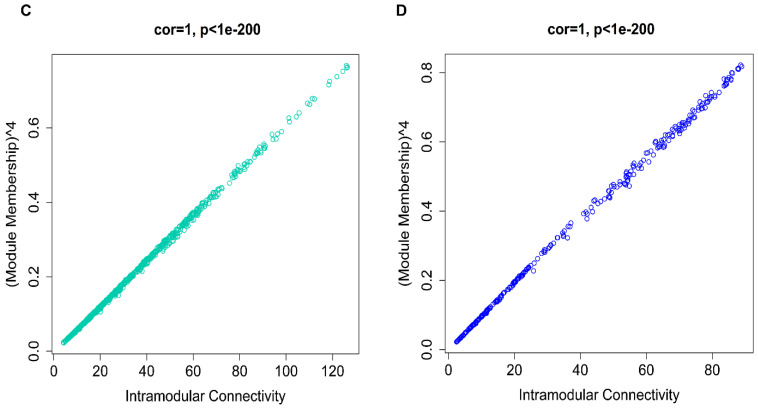
(**A**) Clustering dendrograms (hierarchical) of non-noisy HCC-specific DEGs clustered based on dissTOM, showcasing two modules (i.e., blue and turquoise) obtained using DTC. (**B**) WGCN is represented as a TOM plot wherein module assignments, along with clustered gene dendrograms (hierarchical), are showcased at the top and left panel. Dark-shaded blocks along the diagonal signify the identified modules. Scatterplots exhibiting the significant correlation of k.in with MM across (**C**) turquoise and (**D**) blue modules.

Post noisy DEGs and sample outliers’ check, we input a total of 1059 *HCC*-specific DEGs corresponding to 413 samples for WGCN establishment. The WGCN was constructed at β=4 (corresponding to $R^{2}=0.81$). Clustering dendrogram (hierarchical) and DTC algorithm resulted in a total of two modules (i.e., blue and turquoise) as shown in Figure 3A. The TOM plot for these modules, represented as a heatmap, is shown in Figure 3B. Figure 3C,D depicts the scatterplots showing a significant correlation between k.in and MM for both these modules. As evidenced, since both these modules were having an equal correlation (i.e., r=1), thereby we considered both these modules as hub modules. A total of 47 and 11 hub DEGs were obtained in blue and turquoise hub modules with MM values exceeding 0.9.

### 2.3. PPIN Construction and Modular Analysis

**Figure 4:**
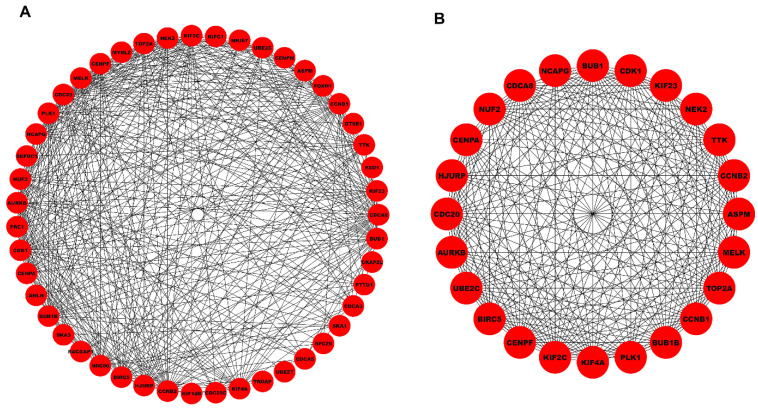
(**A**) Unweighted and undirected PPIN comprising 47 nodes and 387 edges (corresponding to an interaction score>0.4). (**B**) Top-scoring PPIN cluster comprising 24 nodes and 239 edges. Red-colored nodes signify the overexpression status of DEGs.

All 58 hub DEGs were given as an input to the STRING database and the established PPIN (corresponding to an interaction score>0.4) comprised 47 nodes and 387 edges as shown in Figure 4A. The top-scoring PPIN cluster comprised 24 nodes and 239 edges as shown in Figure 4B.

### 2.4. Univariate OS and Pathway Enrichment Analyses

**Figure 5:**
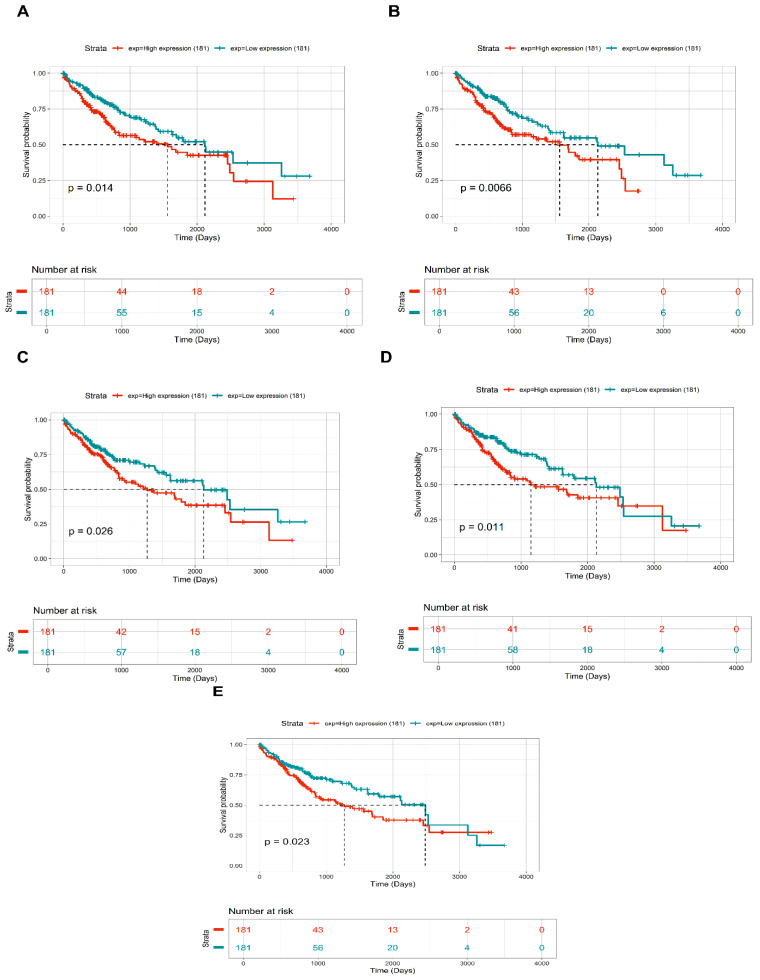
KM plots showcasing the OS in case of (**A**) TTK, (**B**) CENPA, (**C**) NUF2, (**D**) KIF2C, (**E**) CDCA8 across 363 tumor patient samples. Low and high expression groups are represented by cyan and red colors.

**Figure 6:**
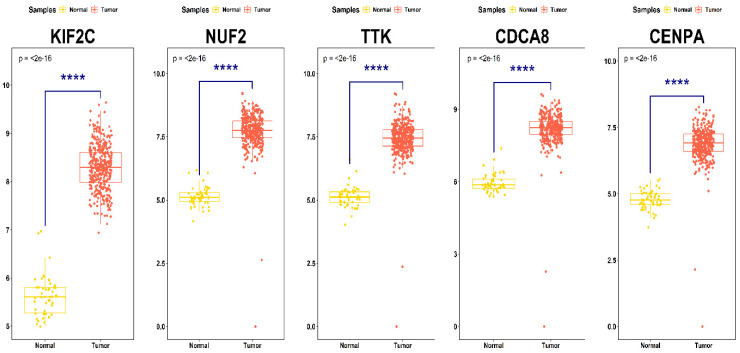
Box-and-whisker plots showing expression intensity distribution of KIF2C, NUF2, TTK, CDCA8, and CENPA across normal and tumor patient samples. Horizontal lines within the boxes represent the median values while minimum and maximum values label the axes endpoints. *p*-values shown at the top of boxplots represent significance levels between sample groups for each prognostic DEGs. **** stands for *p*-value < 0.0001.

**Figure 7:**
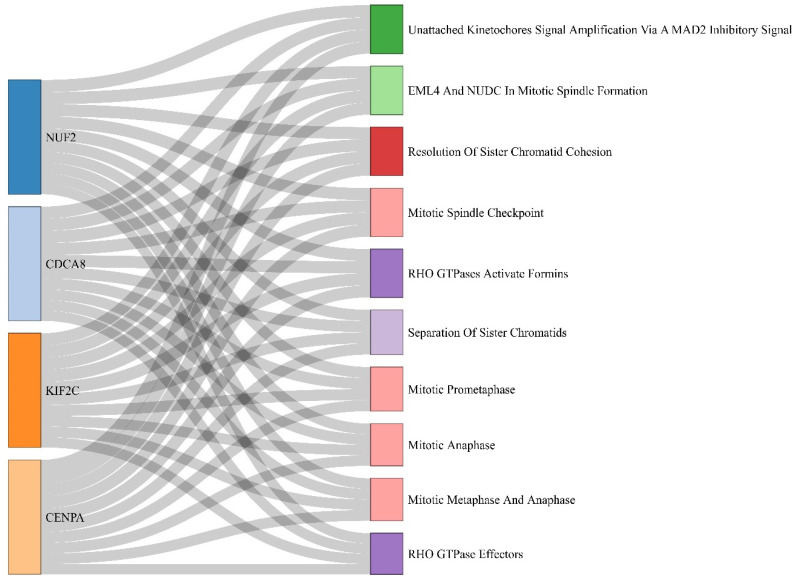
Sankey plot showing the association of the top 10 most significant pathways with corresponding four prognostically significant PPIN cluster DEGs.

Based on the threshold for OS analysis, TTK, CENPA, NUF2, KIF2C, and CDCA8 showed significant differences between high- and low-expression cohorts among all PPIN cluster DEGs. KM plots showing significant OS of these DEGs across HCC patient samples are shown in Figure 5A–E. As noted, higher mRNA expression levels of all these DEGs correlated with poor OS of HCC patients. Box-and-whisker boxplots showing the relative expression distribution of all these prognostic DEGs with respect to tumor and normal samples are shown in Figure 6. Sankey plot showing the association of the top 10 most significant pathways with corresponding four prognostically significant PPIN cluster DEGs are shown in Figure 7. The topmost significant pathway was unattached kinetochores signal amplification via a MAD2 inhibitory signal (BH-*p*-value = $3.77\times 10^{-8}$).

### 2.5. Mutational Analysis of Prognostically Significant DEGs

**Figure 8:**
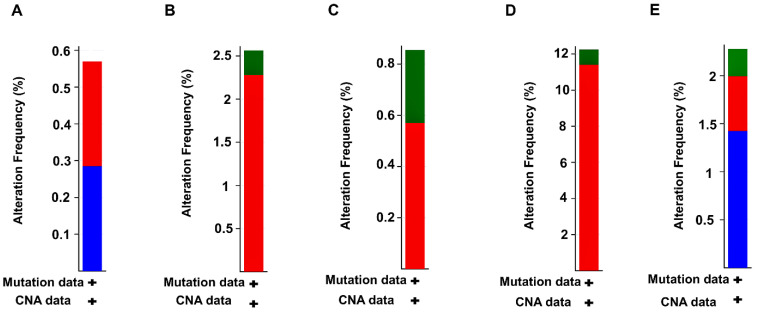
Barplots showing alteration frequencies of (**A**) KIF2C, (**B**) NUF2, (**C**) TTK, (**D**) CDCA8, (**E**) CENPA across the TCGA-HCC cohort. Red, blue, and green colored shaded areas in barplots correspond to amplifications, deep deletions, and missense mutations.

We selected 363 tumor patient samples from HCC (TCGA, Firehose Legacy) cohort within cBioPortal for mutational analysis of *TTK*, *CENPA*, *NUF2*, *KIF2C*, and *CDCA8*. Altogether, these DEGs indicated an alteration in 60 (~18%) patient samples. *TTK*, *CENPA*, *NUF2*, *KIF2C*, *CDCA8* reported 2.21%, 2.49%, 12.15%, 0.83%, 0.55% mutation frequencies. Barplots shown in Figure 8A–E represent overall alteration frequencies of *TTK*, *CENPA*, *NUF2*, *KIF2C*, and *CDCA8* based on the cancer-type summary analysis. 0.28%, 0.83%, 0.28%, 0.28% missense mutation frequencies were reported for *KIF2C*, *NUF2*, *TTK*, *CENPA*. 0.55%, 11.33%, 0.55%, 0.28%, 2.21% amplification frequencies were reported for *KIF2C*, *NUF2*, *TTK*, *CDCA8*, and *CENPA*. The 1.38% and 0.28% deep deletion frequencies were reported for *TTK* and *CDCA8*.

### 2.6. Tumor Immune Infiltration Analysis

**Figure 9:**
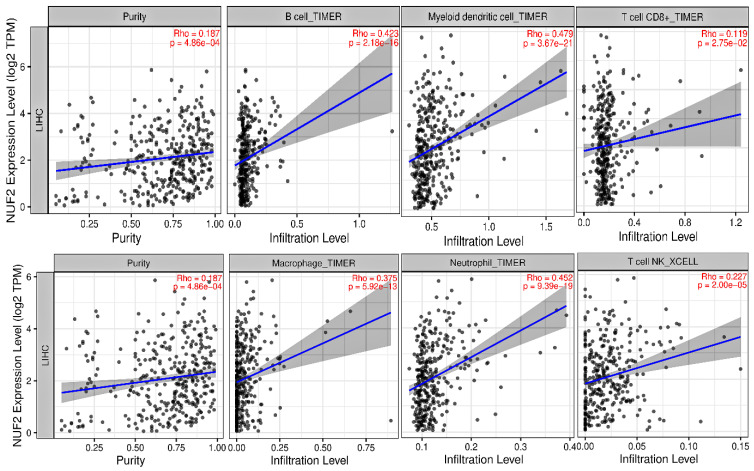
Scatterplots showing significant correlations of NUF2 with B cells, mDCs, ${CD8}^{+}$ T cells, MPs, neutrophils, and NKT cells across the TCGA-HCC cohort.

Scatterplots in Figure 9 shows the correlation of *NUF2* with tumor purity along with B cells, DCs, ${CD8}^{+}$ T cells, MPs, neutrophils, and NKT cells across the TCGA-HCC cohort. *NUF2* reported significant positive correlations with B cells (r=0.423, *p*-value $=2.18\times 10^{-16}$), mDCs (r=0.479, *p*-value $=3.67\times 10^{-21}$), ${CD8}^{+}$ T cells (r=0.119, *p*-value $=2.75\times 10^{-2}$), MPs (r=0.375, *p*-value $=5.92\times 10^{-13}$), neutrophils (r=0.452, *p*-value $=9.39\times 10^{-19}$), NKT cells (r=0.227, *p*-value $=2\times 10^{-5}$). Also, *NUF2* reported a significant positive correlation with tumor purity (r=0.187, *p*-value $=4.86\times 10^{-4}$) across the TCGA-HCC cohort.

## 3. Discussion

In this study, we employed an integrative “funnel” strategy to transition from broad transcriptomic dysregulation to highly specific prognostic markers. By sequentially applying filters for co-expression stability, protein interactome density, and clinical survival significance, we identified five hub genes (TTK, CENPA, NUF2, KIF2C, and CDCA8) that appear to be critical to HCC progression. While statistical correlation does not inherently confirm biological causation, the convergence of these independent analytical streams suggests that these genes are not merely “passenger” alterations but are high-priority candidates with strong evidence for central roles in the HCC mitotic landscape [18,19].

We employed WGCNA, a systems biology technique commonly used to identify biologically significant gene modules in cancer transcriptomic datasets, to capture coordinated gene regulation rather than isolated expression variations [9]. WGCNA has been successfully used in several earlier studies to identify prognostic gene modules in HCC, which are often enriched for chromosome segregation, DNA replication, and cell cycle regulation [20]. In agreement with these reports, our analysis identified two hub modules exhibiting strong correlations between module membership and intramodular connectivity, suggesting the presence of highly interconnected and functionally relevant gene clusters.

A core cluster of genes linked to mitosis was identified after further refinement using modular analysis and the PPIN. analysis. Elevated expression of *TTK*, *CENPA*, *NUF2*, *KIF2C*, and *CDCA8* was strongly associated with poor OS in HCC patients, as determined by univariate survival analysis. Similar findings have been documented in earlier bioinformatics research, where aggressive tumor behavior and poor clinical outcomes were linked to overexpression of cell cycle regulators and mitotic checkpoint genes [20,21]. These findings collectively reinforce the critical role of mitotic dysregulation in HCC pathogenesis.

These prognostically relevant genes were primarily involved in kinetochore signaling and mitotic checkpoint pathways, including unattached kinetochore signal amplification, according to pathway enrichment analysis. Chromosome instability, a characteristic of HCC that contributes to tumor heterogeneity and development, is known to be promoted by dysregulation of these pathways [18]. We acknowledge that the topology of biological networks is sensitive to the choice of interactomes and scoring thresholds. However, our enrichment analysis demonstrated that these five hub genes are functionally synchronized within the mitotic checkpoint and kinetochore signaling pathways. This biological coherence provides a layer of validation that transcends individual network parameters. Furthermore, the consistency of these genes as central nodes across varied modularity settings reinforces their potential as robust prognostic biomarkers and viable therapeutic targets in HCC.

About half of the tumor samples showed genomic changes, according to mutational analysis, with *NUF2* showing relatively higher mutation frequencies, mostly due to gene amplifications. Similar patterns of copy number abnormalities affecting cell cycle genes have been previously identified in HCC, despite the relatively low individual mutation rates. These variations are believed to lead to aberrant mitotic activity and tumor formation [19].

Lastly, *NUF2* expression was found to be significantly positively correlated with several immune cell groups, including B cells, DCs, T cells, MPs, neutrophils, and NKT cells, according to immune infiltration analysis. These results are consistent with growing evidence that dysregulated cell cycle genes may affect immune cell recruitment and the TME. Comparable associations between prognostic hub gene expression and immune infiltration have also been reported in previous HCC studies integrating immune deconvolution analyses [22].

When combined, our findings are in line with previous research and add to our understanding of transcriptome dysregulation, co-expression networks, protein interactions, survival outcomes, genetic changes, and immune infiltration patterns. This thorough approach emphasizes the critical role of mitotic checkpoint dysregulation in disease progression and identifies *TTK*, *CENPA*, *NUF2*, *KIF2C*, and *CDCA8* as important prognostic drivers in HCC.

## 4. Materials and Methods

### 4.1. mRNA-Seq Data Extraction and DEA

To investigate gene expression differences between normal and tumor tissues, we utilized publicly available RNA-sequencing data from the TCGA-HCC cohort, accessed through the UCSC Xena browser [23] (https://xenabrowser.net/). We focused on mRNA counts generated using the Illumina HiSeq platform (Illumina Inc., San Diego, CA, USA). Only those patient samples were retained for which survival data was present. Primarily, we back-log-transformed the raw original counts to obtain equivalent integer values across both normal and tumor samples. Next, we deployed the DESeq2 R package [24] to normalize the data using VST and achieve ${log}_{2}$-transformed expression values. Batch effects were corrected utilizing the ARSyNseq function within the NOISeq R package [25,26]. Finally, gene identifiers were converted from Ensembl IDs to their corresponding HGNC symbols using the biomaRt R package [27,28] wherein only protein-coding genes were retained for further analysis. Genes with multiple Ensembl IDs were averaged to avoid redundancy. HCC-specific DEGs were identified utilizing the Limma R package [29] corresponding to a *p*-value <0.05 and $\left|{log}_{2}(fold\;change)\right|>1.5$.

### 4.2. Non-Trait-Based WGCN Construction and Hub Module/Genes Selection

All HCC-specific DEGs were passed to Pigengene R package [30] for eradicating any noisy DEGs. Also, any possible sample outliers were checked and removed before proceeding with WGCN establishment. The protocol in sequence for WGCN formation, as per the appropriate β with respect to SFT, ME/MEdiss computation, and module assignment, was performed as discussed previously [31,32]. Modules with the highest correlation values between MM and k.in were regarded as hub modules. The hub DEGs from the hub module(s) with MM>0.9 were retained for further analysis.

### 4.3. PPIN Construction and Modular Analysis

All the hub DEGs were given as an input to the STRING v12.0 database [33] in order to construct a PPIN corresponding to medium confidence (i.e., interaction score>0.4) and afterwards visualized via Cytoscape v3.10.3 [34]. PPIN cluster was obtained utilizing the MCODE app v 2.0.2 [35] with settings as discussed previously [36].

### 4.4. Univariate OS and Pathway Enrichment Analyses

The PPIN cluster DEGs expression values were bifurcated into high and low expression groups based on whether expression≥median or expression<median. Log-rank *p*-value < 0.05 was considered a statistically significant threshold for prognostic assessment. Also, we ensured that the prognostically significant DEGs’ survival curves matched their expression levels. We input all prognostically significant DEGs to Enrichr database [37,38] wherein we choose the Reactome library for compiling the top 10 most significant (BH-*p*-value < 0.05) pathways.

### 4.5. Mutational Analysis of Prognostically Significant DEGs

We accessed the cBioPortal [39,40] (https://www.cbioportal.org/) database to investigate the genomic alterations in prognostically significant PPIN cluster DEGs. We selected the HCC (TCGA, Firehose Legacy) cohort in cBioPortal and matched with the same tumor samples used initially for DEA.

### 4.6. Tumor Immune Infiltration Analysis

TIMER web-based tool [41,42] (https://compbio.cn/timer3/, accessed on 29 April 2026) was queried to discover the correlation between prognostically significant highly mutated DEGs expression levels and B cells, DCs, ${CD8}^{+}$ T cells, MPs, neutrophils, and NKT cells across the TCGA-HCC cohort.

## 5. Conclusions

In conclusion, *TTK*, *CENPA*, *NUF2*, *KIF2C*, and *CDCA8* were found to be important prognostic hub genes in HCC by this integrated bioinformatics analysis. These genes are prognostically relevant, exhibit significant genomic and immunological correlations, and are closely linked to cell cycle and mitotic checkpoint pathways. In addition to suggesting prospective biomarkers and therapeutic targets that require further experimental and clinical confirmation, our findings provide insights into the molecular pathways underlying HCC evolution.

## Data Availability

The original contributions presented in this study are included in the article. Further inquiries can be directed to the corresponding author.

## Abbreviations

HCC, Hepatocellular carcinoma; RNA-seq, RNA sequencing; DEA, differential expression analysis; TCGA, the cancer genome atlas; WGCNA, weighted gene co-expression network analysis; PPIN, protein–protein interaction network; OS, overall survival; mRNA, messenger RNA; DEGs, differentially expressed genes; WGCN, weighted gene co-expression network; SFT, scale-free topology; MM, module membership; k.in, intramodular connectivity; ME, module eigengene; MEdiss, module eigengene dissimilarity; STRING, Search Tool for the Retrieval of Interacting Genes/Proteins; MCODE, molecular complex detection; MP, macrophage; NKT, natural killer T; DC, dendritic cell; TME, tumor microenvironment.
